# Educational videos vs interactive e-modules: adolescents’ menstrual hygiene knowledge, attitudes, and practices

**DOI:** 10.1016/j.xagr.2026.100699

**Published:** 2026-09-26

**Authors:** Josi Noviani, Dwi Prasetyo, Dini Pusianawati, Qorinah Estiningtyas Sakilah Adnani, Dzulfikar Djalil Lukmanul Hakim, Setyorini Irianti, Isabella Garti

**Affiliations:** 1 Master’s Program in Midwifery, Faculty of Medicine, Universitas Padjadjaran, Bandung, West Java, Indonesia (Noviani); 2 Department of Child Health, Faculty of Medicine, Padjadjaran University, Dr. Hasan Sadikin General Hospital, Bandung, West Java, Indonesia (Prasetyo, Hakim); 3 Department of Obstetric and Gynaecology, Universitas Padjadjaran, Bandung, West Java, Indonesia (Pusianawati and Irianti); 4 Department of Public Health, Faculty of Medicine, Universitas Padjadjaran, Bandung, West Java, Indonesia (Adnani); 5 Midwifery Working Group, Center for Health System Studies and Health Workforce Education Innovation, Faculty of Medicine, Universitas Padjadjaran, Bandung, West Java, Indonesia (Adnani); 6 Molly Wardaguga Institute for First Nations Birth Rights, Faculty of Health Charles Darwin University, Brinkin, Northern Territory, Australia (Garti)

**Keywords:** adolescent girls, educational video, health education, interactive e-module, menstrual hygiene

## Abstract

**BACKGROUND:**

Poor menstrual hygiene knowledge and practices among adolescent girls remain a major public health concern in resource-limited settings, where access to comprehensive reproductive health education is often limited.

**OBJECTIVE:**

To evaluate the effectiveness of interactive e-modules against educational videos in improving menstrual hygiene knowledge, attitudes, and practices among adolescent females.

**STUDY DESIGN:**

A quasi-experimental pretest-posttest two-group design was conducted at a public junior high school in the Pulau Punjung District of Indonesia. Seventy adolescent girls were recruited via purposive selection and assigned to either an interactive e-module group or a video group (*n*=35 each group). Knowledge, attitudes, and practices were assessed utilizing validated questionnaires. The data analysis comprised the Wilcoxon signed-rank test and the Mann–Whitney *U* test, with effectiveness evaluated by N-gain scores.

**RESULTS:**

Both interventions led to substantial improvements in knowledge, attitudes, and practices (*P*<.001). The interactive e-module demonstrated greater effectiveness than the video intervention across all areas, including knowledge (*P*<.001), attitudes (*P*=.022), and practices (*P*<.001). Elevated N-gain scores further validated the interactive e-module’s superiority.

**CONCLUSION:**

Interactive e-modules surpass educational videos in enhancing menstrual hygiene knowledge, attitudes, and practices among adolescent girls, suggesting that they may be a preferred strategy for delivering menstrual health education in schools and other adolescent health programs.

**AJOG Global Reports at a Glance**

**Why was this study conducted?**

To evaluate the effectiveness of two digital learning approaches, interactive e-modules and educational videos, in improving menstrual hygiene knowledge, attitudes, and practices among adolescent girls in Indonesia.

**Key findings**

Interactive e-modules improved knowledge of menstrual hygiene more than videos. Both digital interventions significantly improved menstrual hygiene knowledge, attitudes, and practices. Interactive digital learning can strengthen school-based menstrual health education.

**What does this add to what is known?**

This study provides evidence from Indonesia that digital menstrual health education is an effective approach for improving menstrual hygiene outcomes among adolescent girls. It demonstrates that interactive e-modules yield greater knowledge gains than video-based education, supporting the integration of engaging digital learning strategies into school health programs in low- and middle-income settings.

## Introduction

Adolescence is a transitional phase between childhood and adulthood, spanning ages 10 to 19.^1^ This period is essential for setting the groundwork for long-term health. At this stage, teenage girls undergo swift pubertal maturation, marked by the commencement of menstruation.^2^ In the female reproductive system, menstruation is a typical physiological process driven by coordinated hormonal changes, and it occurs from menarche to menopause.^3^ Menstruation is not only a biological process but is also intricately linked to reproductive health, quality of life, comfort, and women’s productivity in daily tasks.^3^^,^^4^

More than 300 million women worldwide menstruate on a daily basis.^5^ However, menstrual education in schools remains unavailable in most schools worldwide.^6^ Globally, approximately 500 million women face limited access to menstrual products and adequate hygiene facilities.^5^ This situation reflects period poverty, which includes limited access to menstrual products, inadequate sanitation facilities, limited knowledge, and social stigma surrounding menstruation.^6^, ^7^, ^8^, ^9^ These conditions can affect reproductive health, increase the risk of infection, contribute to school absenteeism, and cause shame and low self-esteem among adolescent girls.^7^ Menstrual hygiene involves using clean menstrual products, regularly changing menstrual pads, and understanding the menstrual cycle and its management. Poor menstrual hygiene practices can increase the risk of urinary tract infections, bacterial vaginosis, genital irritation, and itching associated with improper use of menstrual pads.^9^ Therefore, menstrual hygiene education is an important component of efforts to maintain reproductive health among adolescent girls.

In Indonesia, adolescent girls represent a large population group. In 2026, Indonesia’s population is projected to reach 287.2 million, with girls aged 10 to 19 years accounting for approximately 15.24% of the total female population.^10^ However, menstrual hygiene practices among adolescents remain a serious challenge. A multi-country study involving Indonesia showed that access to accurate information on menstrual hygiene remains limited.^11^ In general, knowledge, attitudes, and menstrual hygiene practices are closely interrelated, with access to health information playing a key role in shaping these outcomes, and Indonesia is no exception.^2^^,^^7^ Indonesian studies have also shown persistent gaps in knowledge, attitudes, and menstrual hygiene practices among adolescent girls. A study in Yogyakarta found that most female students had moderate knowledge, but all respondents showed unfavorable attitudes toward menstrual hygiene.^12^ Another study in Tangerang Regency found that nearly half of adolescent girls still practiced poor menstrual hygiene.^13^ Meanwhile, in Batanghari Regency, Jambi, more than half of adolescent girls had low knowledge and inadequate menstrual hygiene practices.^14^ Similarly, in Dharmasraya Regency, West Sumatra, preliminary findings indicated poor menstrual hygiene practices, reluctance to discuss menstruation, and reliance on unverified online sources for menstrual health information.

Health education can improve menstrual hygiene knowledge, attitudes, and practices among adolescent girls,^7^^,^^15^ with digital approaches being more accessible than conventional methods. Interactive e-modules and educational videos have both demonstrated effectiveness.^16^, ^17^, ^18^ However, evidence directly comparing the effectiveness of interactive e-modules and educational videos remains limited. While numerous studies have evaluated menstrual hygiene education interventions, few have compared the effects of these two digital learning approaches on adolescent girls’ knowledge, attitudes, and menstrual hygiene practices. This study addresses this gap by comparing interactive e-modules with educational videos for menstrual hygiene education among adolescent girls at a public junior high school in Pulau Punjung District, Dharmasraya Regency, West Sumatra.

## Materials and methods

### Study design and setting

This quasi-experimental study used a pretest-posttest two-group design. It was conducted at a public junior high school in Pulau Punjung District, Dharmasraya Regency, West Sumatra, Indonesia, in March 2026. Dharmasraya Regency, West Sumatra, was selected due to limited access to reproductive health education among younger adolescents, with preliminary findings indicating poor menstrual hygiene practices and reliance on unverified online information. The school was purposively selected based on a preliminary study showing that female students had not previously received specific menstrual hygiene education. The site was also selected based on the feasibility of conducting the study and the availability of digital infrastructure to support technology-based educational interventions.

### Population and sample

The study population consisted of adolescent girls in grades 7 and 8 at a public junior high school in Pulau Punjung District, Dharmasraya Regency. A total of 70 adolescent girls were selected using purposive sampling. The sample size was calculated using the formula for comparing two means:

$$\frac{n=\;\;\;2S^{2}{\left(Z\alpha +Z\beta \right)}^{2}}{d^{2}}$$

Details:

*n*: sample size per group
*Zα: Z* value at a 95% confidence level
*Zβ: Z* value at 90% power
*d*: expected mean difference between the two groups
*S*: pooled standard deviation

Based on the calculation and allowing for potential dropout, each group included 35 adolescent girls, resulting in a total sample of 70 participants. The participants were divided into the interactive e-module group and the educational video group.

### Intervention: development and validation of digital media

The study used two types of digital media: an interactive e-module and an educational video. The media were developed using the 4D model (Define, Design, Develop, and Disseminate) to ensure validity and practicality for adolescent girls. During the Define stage, needs were identified based on a preliminary study highlighting the need for innovative menstrual hygiene education. The Design stage produced the interactive e-module, while the comparison group received an educational video from UNICEF Indonesia. Both media were integrated into a mobile application with personal accounts, enabling objective monitoring via a digital tracking system. The educational content delivered through both the interactive e-module and the standard educational video was identical, covering the same menstrual hygiene topics. In the Develop stage, the media were validated by three experts in content, media, and language using a 4-point Likert scale. Validation results indicated high feasibility: content scores of 92.5% and 97.5%, language 93.2%, and media 80%. A pilot test was conducted with 10 adolescent girls with characteristics similar to those of the target population to ensure functionality and engagement before full implementation.

### Data collection and instrument

Data were collected using a structured questionnaire covering three domains: knowledge (20 items), attitudes (20 items), and practices (16 items) related to menstrual hygiene (Supplemental File 1). Validity was tested on 30 adolescent girls outside the study sample using Pearson correlation (all items ≥0.361, *P*<.05), and reliability was confirmed with Cronbach’s alpha ≥0.77 for all variables.

### Ethical considerations

Ethical approval for this study was obtained from the Research Ethics Committee Universitas Padjadjaran Bandung, prior to participant recruitment (Approval No: 140/UN6.KEP/EC/2026). Permission to conduct the study was also obtained from the participating schools and relevant education authorities. Participation was voluntary, and written informed consent was obtained from participants aged 18 years. For participants younger than 18 years, written parental or guardian consent and participant assent were obtained before enrolment. Participants were informed of the study objectives, procedures, potential risks and benefits, and their right to decline participation or withdraw at any stage without penalty. No personally identifying information was included in the exported dataset, and all responses were deidentified prior to analysis. Data were stored securely on password-protected institutional servers and were accessible only to members of the research team. As the intervention involved menstrual health education, participants were advised that they could omit any question they were uncomfortable answering. The study posed minimal risk, and all procedures were conducted in accordance with the ethical principles of the Declaration of Helsinki and applicable national research ethics guidelines. Participant confidentiality and privacy were maintained throughout the study.

### Data collection procedure

Data were collected in three main phases: pretest, intervention, and post-test. In the pretest phase, participants completed the questionnaire in the classroom under the direct supervision of the researchers and a classroom teacher to ensure the authenticity of responses. During the 2-week intervention phase, participants were divided into two groups: the interactive e-module group and the educational video group. They accessed the mobile application independently via their smartphones, and compliance was monitored through the app’s digital tracking system to ensure adequate exposure. Both groups received their assigned digital intervention for 2 weeks. The post-test was conducted in the classroom 2 weeks after completion of the intervention period, using the same instrument under the same supervision, allowing evaluation of knowledge adoption and minimizing memory bias.

### Data analysis

Data were analyzed using SPSS version 31. The Shapiro–Wilk test was used to assess the normality of the data distribution. As the results indicated a non-normal distribution, nonparametric tests were applied. Within-group comparisons (pre- and postintervention) were analyzed using the Wilcoxon signed-rank test, while between-group comparisons were performed using the Mann–Whitney *U* test to evaluate differences in effectiveness between the interactive e-module and educational video groups. Additionally, N-gain scores were calculated to determine the magnitude of improvement for each variable. Statistical significance was set at *P*<.05.

## Results

Table 1 presents the baseline characteristics, menstrual history, and initial information sources of the participants. Both groups included 35 participants and were comparable in grade, age, age at menarche, and duration of menstruation (*P*>.05). These findings indicate that the groups were homogeneous at baseline prior to the intervention.

**Table 1:** Baseline characteristics and menstrual history of participants in the interactive e-module and video groups

Characteristic	Interactive e-module group *f* (%)	Video group *f* (%)	*P* value
Grade			
7	17 (48.6%)	17 (48.6%)	1.000
8	18 (51.4%)	18 (51.4%)	
Current age			
12–14 y	21 (60.0%)	22 (62.9%)	.806
14–15 y	14 (40.0%)	13 (37.1%)	
Age at menarche			
<12 y	8 (22.9%)	7 (20.0%)	.771
12–13 y	27 (77.1%)	28 (80.0%)	
Duration of menstruation			
<7 d	21 (60.0%)	25 (71.4%)	.475
7 d	9 (25.7%)	5 (14.3%)	
>7 d	5 (14.3%)	5 (14.3%)	

### Educational media access

Participants accessed the interactive e-module more frequently than the video. The highest frequency of access was 4 times for the e-module and 2 times for the video. Notably, repetitions up to 9 occurred exclusively in the e-module group (Figure). The total number of accesses was higher in the interactive e-module group (170 accesses) compared with the video group (102 accesses). This difference indicates that participants in the e-module group revisited the learning materials more frequently during the intervention period.

**Figure:**
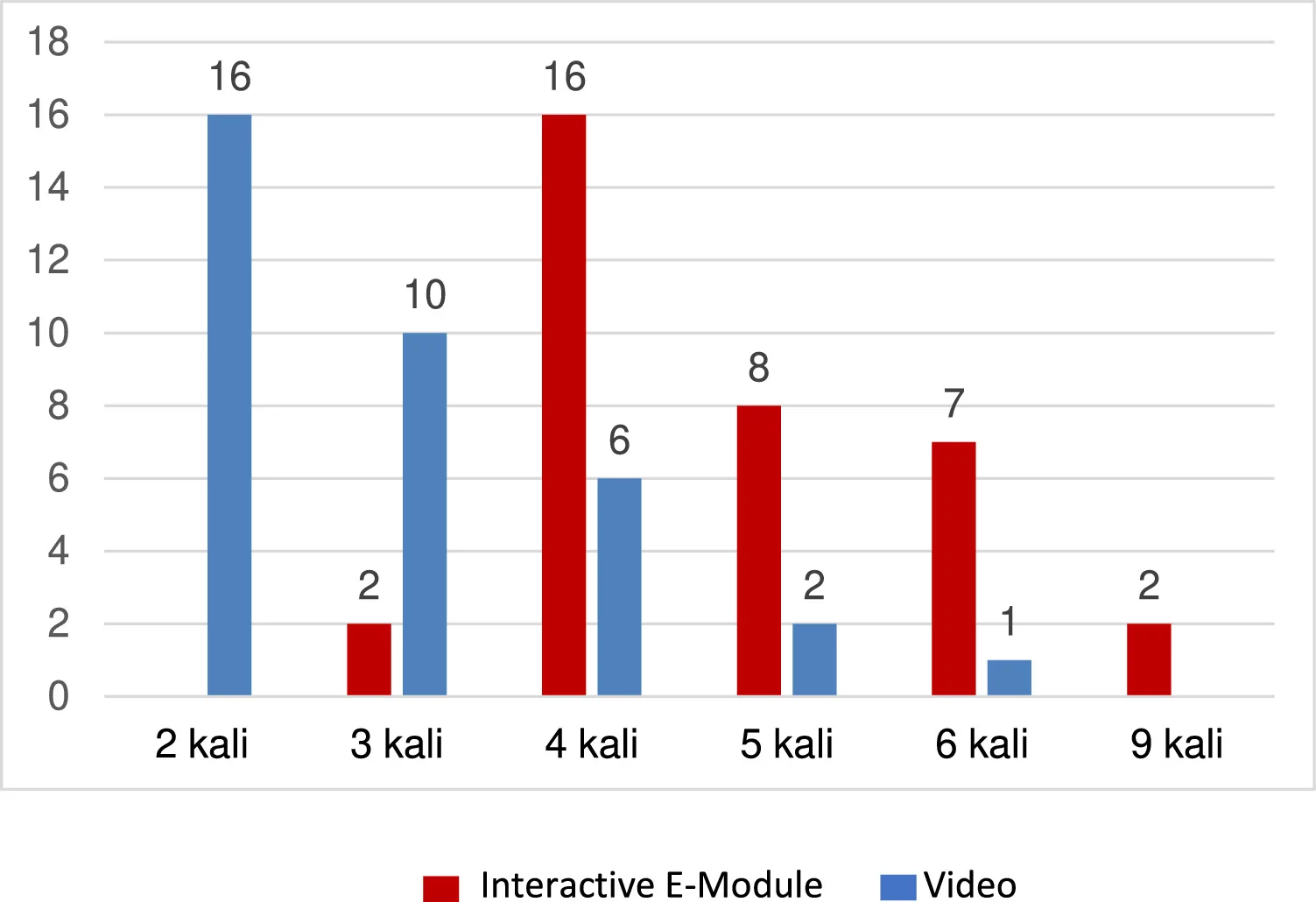
Distribution of access frequency for the interactive e-module and video

Noviani. Educational videos vs interactive e-modules. Am J Obstet Gynecol 2026.

### Interactive e-module

In the interactive e-module group, pretest scores for knowledge, attitudes, and menstrual hygiene practices were 50, 72, and 50, respectively. These scores increased to 95, 88, and 93.75 following the health education intervention. Median scores in all domains demonstrated statistically significant improvements after the intervention (Wilcoxon signed-rank test, *P*<.05) (Table 2).

**Table 2:** Differences in menstrual hygiene knowledge, attitudes, and practices before and after the interactive e-module intervention

	Before intervention		After intervention		*P* value
Variable	Median	Min–max	Median	Min–max	
Knowledge	50	35–65	95	85–100	<.001
Attitude	72	62–80	88	73–99	<.001
Practice	50	31–75	93.75	50–113	<.001

Based on the Wilcoxon signed-rank test.

### Videos

In the video group, pretest scores for knowledge, attitudes, and menstrual hygiene practices were 50, 72, and 56.25, respectively. These scores increased to 90, 84, and 68.75 after the health education intervention. Median scores in all domains showed statistically significant improvements following the intervention (Wilcoxon signed-rank test, *P*<.05) (Table 3).

**Table 3:** Differences in menstrual hygiene knowledge, attitudes, and practices before and after the video intervention

	Before intervention		After intervention		*P* value
Variable	Median	Min–max	Median	Min–max	
Knowledge	50	25–65	90	70–100	<.001
Attitude	72	61–81	84	78–95	<.001
Practice	56.25	38–63	68.75	56–81	<.001

Based on the Wilcoxon signed-rank test.

### Interactive e-module vs video

The Mann–Whitney *U* test revealed significant differences between the interactive e-module and video groups in knowledge (*P*<.001), attitudes (*P*=.022), and practices (*P*<.001). Based on N-gain scores, the interactive e-module group demonstrated greater improvements in knowledge, attitudes, and menstrual hygiene practices than the video group (Table 4).

**Table 4:** Differences in the effects of the interactive e-module and video media on knowledge, attitudes, and menstrual hygiene practices

Variable	Interactive e-module (N-gain score)	Video (N-gain score)	*P* value
Knowledge	90	80	<.001
Attitude	58.06	46.43	.022
Practice	87.50	42.86	<.001

Based on the Mann–Whitney *U* test.

## Discussion

### Principal findings

The majority of respondents were aged 12 to 15 years, corresponding to early adolescence, a developmental stage characterized by rapid cognitive, emotional, and social development and an increased capacity to understand complex health information.^1^ Most respondents reported experiencing menarche between 12 and 13 years of age, consistent with the reported global age range of 10 to 16 years.^19^ Additionally, most participants reported a menstrual duration of fewer than 7 days, which falls within the normal physiological range. Menstrual duration has practical implications for menstrual hygiene management, as longer periods may increase the need for appropriate menstrual hygiene practices and resources.^20^ Baseline characteristics, including grade level and other demographic variables, were comparable between groups (all *P*>.05), indicating that the groups were well balanced before the intervention and suitable for comparison.^15^

### Results

Against this background, this study compared the effectiveness of an interactive e-module and an educational video in improving adolescent girls’ menstrual hygiene knowledge, attitudes, and practices. Both the interactive e-module and educational video improved adolescents’ menstrual hygiene knowledge, attitudes, and practices. However, the interactive e-module intervention markedly enhanced knowledge, attitudes, and practices about menstrual hygiene (*P*<.001). Interactive interventions are particularly engaging for adolescents, and e-modules facilitate learning by combining text, graphics, animations, and interactive components to enhance comprehension.^18^^,^^21^^,^^22^ Our findings suggest that digital media may enhance health literacy among adolescent girls through its engaging and easy-to-understand format. Given that adolescents are digital natives and among the most frequent smartphone and social media users globally, digital platforms represent an appealing and effective medium for health education.^23^ Furthermore, in the context of reproductive health education, digital media mitigates psychological obstacles, including embarrassment.^23^ Additionally, digital interventions promote more positive perceptions of menstrual health,^24^ which are likely to influence behavioral change. Consistent with this, adolescents in our study who received the interactive e-module reported significantly better menstrual hygiene practices than those in the video intervention group.

The video intervention also enhanced knowledge, attitudes, and practices regarding menstrual hygiene (*P*<.001), consistent with previous studies demonstrating that video-based education, when combined with complementary educational resources such as detailed handouts and PowerPoint presentations, can effectively improve menstrual health literacy in school settings.^25^^,^^26^ Video-based learning is therefore a practical alternative for menstrual health education, particularly in resource-constrained settings where interactive e-modules are not feasible. Videos can provide engaging, standardized educational content that is easy to disseminate across schools, making them a valuable strategy for improving menstrual health literacy.^27^

The comparison of groups demonstrated that the interactive e-module outperformed the video intervention, as evidenced by superior N-gain scores across all variables and statistically significant differences between groups (*P*<.05). The results demonstrate that while both interventions were effective, interactive e-modules may offer greater educational benefits by promoting active engagement and knowledge acquisition. Students can manage their learning, revisit lessons, and receive prompt feedback, thereby increasing engagement.^24^^,^^28^ Quiz functionalities, prompt feedback, and autonomous navigation also enhance student engagement.^28^

### Implications

As digital technology increasingly integrates into adolescents’ daily lives, digital learning offers a valuable opportunity to strengthen reproductive health education.^29^ Despite growing interest in digital education, evidence directly comparing different digital learning modalities for menstrual health remains scarce. This study contributes to the growing evidence supporting the use of digital interventions for menstrual health education by demonstrating that both interactive e-modules and educational videos are effective in improving menstrual hygiene knowledge, attitudes, and practices. These findings suggest that interactive digital learning should be considered in the design of school-based menstrual health education programs to enhance adolescent health literacy and support informed menstrual hygiene practices. Incorporating interactive digital learning into school-based menstrual health education may strengthen adolescent health literacy and equip young people with the knowledge and skills needed to adopt informed menstrual hygiene practices.

### Strengths and limitations

A key strength of this study is the use of a quasi-experimental pretest–posttest two-group design, which enabled comparison of two digital educational approaches. The use of a structured questionnaire assessing knowledge, attitudes, and practices provided a comprehensive evaluation of the intervention outcomes.

This study has several limitations. The quasi-experimental design without random allocation may have introduced selection bias. Outcomes were measured using self-reported questionnaires, which are subject to recall and social desirability bias. In addition, the short follow-up period did not allow for the assessment of long-term knowledge retention or sustained changes in menstrual hygiene practices. Furthermore, differences in access frequency between the interactive e-module and educational video groups during the same intervention period may have influenced learning outcomes. Although access frequency was monitored through the digital tracking system, this study did not assess the association between access frequency and knowledge improvement. Detailed information regarding repeated access to specific sections of the e-module was not collected. Future studies should control access frequency and ensure equivalent content structures between digital learning modalities to better determine whether the effectiveness of interactive e-modules is attributable to their interactive features rather than differences in exposure or content volume. Finally, the study was conducted in a single regency, which may limit the generalizability of the findings to other adolescent populations.

## Conclusion

Digital media interventions, including interactive e-modules and videos, markedly enhanced menstrual hygiene knowledge, attitudes, and practices among adolescent females. The interactive e-module proved more effective than the video across all domains. The interactive and self-directed learning components of the e-module contribute to its superior efficacy in facilitating behavioral change relative to a passive video-based learning medium. Future educational initiatives should consider integrating interactive digital learning into school-based reproductive health programs to enhance menstrual health literacy and improve menstrual hygiene management among adolescent girls.

## CRediT authorship contribution statement

**Josi Noviani:** Writing – review & editing, Writing – original draft, Methodology, Formal analysis, Conceptualization. **Dwi Prasetyo:** Writing – review & editing, Writing – original draft, Methodology. **Dini Pusianawati:** Writing – review & editing, Writing – original draft, Methodology. **Qorinah Estiningtyas Sakilah Adnani:** Writing – review & editing, Writing – original draft, Methodology. **Dzulfikar Djalil Lukmanul Hakim:** Writing – review & editing, Writing – original draft, Methodology. **Setyorini Irianti:** Writing – review & editing, Writing – original draft, Methodology. **Isabella Garti:** Writing – review & editing.

## Appendix Supplementary materials

Image, application 1

## Footnotes

Supplementary material associated with this article can be found in the online version at doi:10.1016/j.xagr.2026.100699.
