# Supplementary material for: Educational videos vs interactive e-modules: adolescents’ menstrual hygiene knowledge, attitudes, and practices
Source: AJOG Glob Rep. 2026 Sep 26;6(4):100699. doi: 10.1016/j.xagr.2026.100699 (PMC13721087; doi:10.1016/j.xagr.2026.100699)
Supplement: Supplementary file 1 [file mmc1.pdf]

QUESTIONNAIRE

Respondent Information

Please complete the following information based on your personal details.

Name	:	
Class	:	
Age	:	
Mobile Phone Number	:	
Address	:	
Date of Last Menstrual Period	:	
Age at Menarche (First Menstruation)	:	

Please mark (X) in the appropriate box based on your most recent menstrual experience.

1. During your most recent menstrual period, how many days did the menstrual bleeding last?

   ☐ Less than 7 days

   ☐ 7 days

   ☐ More than 7 days

2. Have you ever received information about menstrual health and hygiene?

   ☐ Yes

   ☐ No

3. When did you first receive information about menstrual health and hygiene?

   ☐ Before your first menstrual period

   ☐ After your first menstrual period

   ☐ Never

4. Who are your sources of information about menstruation? You may select more than one.

   ☐ Mother

   ☐ Friends

   ☐ Teacher(s)

   ☐ Print media (books, magazines)

   ☐ Internet/social media

MENSTRUAL HYGIENE KNOWLEDGE QUESTIONNAIRE

Instructions:

- Read each statement carefully and answer based on your knowledge.
- Mark (X) next to the appropriate answer.

1. Menstruation is a natural biological process that occurs after puberty, although its timing may vary from month to month.

   A. True B. False

2. Menstruation occurs because the endometrial lining is shed when fertilization does not occur.

   A. True B. False

3. Menstrual blood is discharged through the vagina.

   A. True B. False

4. Menarche is the first menstrual period experienced by a woman.

   A. True B. False

5. The menstrual cycle is the time from the first day of one menstrual period to the first day of the next period.

   A. True B. False

6. A normal menstrual cycle usually lasts between 21 and 35 days.

   A. True B. False

7. Menstruation usually lasts between two and seven days.

   A. True B. False

8. To maintain hygiene during menstruation, sanitary pads should be changed regularly even if they do not yet feel wet or full.

   A. True B. False

9. Personal hygiene during menstruation is necessary every day, including during the final days of menstruation.

   A. True B. False

10. Hands should be washed before and after changing a sanitary pad, even if the pad does not feel wet or dirty.

    A. True B. False

11. Sanitary pads should be changed every 4 to 6 hours.

    A. True B. False

12. Used sanitary pads should be wrapped in paper or plastic before being disposed of in a trash bin.

    A. True B. False

13. Reusable cloth pads can be washed with soap, rinsed with clean water, and dried in direct sunlight.

    A. True B. False

14. Underwear should be changed every day even if it does not feel dirty or wet.

    A. True B. False

15. Sanitary pads are used during menstruation to absorb blood, prevent leakage, and maintain personal hygiene.

    A. True B. False

16. Two types of sanitary pads are commonly used: disposable sanitary pads and reusable cloth pads.

    A. True B. False

17. Reusable cloth pads should not be washed with hot water.

A. True B. False

18. Exercising during menstruation does not cause severe abdominal pain.

A. True B. False

19. Adolescent girls can continue interacting with others during menstruation.

A. True B. False

20. Adolescent girls can continue participating in school activities on the first day of menstruation.

A. True B. False

MENSTRUAL HYGIENE ATTITUDE QUESTIONNAIRE

Instructions:

- Read each statement carefully and indicate the extent to which you agree or disagree.
- Mark (X) in the appropriate response box.

1. Bathing twice a day during menstruation is important for maintaining personal hygiene.

   ☐ Strongly Agree ☐ Agree ☐ Undecided ☐ Disagree ☐ Strongly Disagree

2. Hair should still be washed during menstruation to keep it clean.

   ☐ Strongly Agree ☐ Agree ☐ Undecided ☐ Disagree ☐ Strongly Disagree

3. Washing hands before and after changing a sanitary pad is important to prevent the spread of germs.

   ☐ Strongly Agree ☐ Agree ☐ Undecided ☐ Disagree ☐ Strongly Disagree

4. Underwear stained with menstrual blood should be changed immediately to remain clean and comfortable.

   ☐ Strongly Agree ☐ Agree ☐ Undecided ☐ Disagree ☐ Strongly Disagree

5. Cotton underwear is more comfortable to wear during menstruation.

   ☐ Strongly Agree ☐ Agree ☐ Undecided ☐ Disagree ☐ Strongly Disagree

6. The correct way to clean the genital area is from front to back.

   ☐ Strongly Agree ☐ Agree ☐ Undecided ☐ Disagree ☐ Strongly Disagree

7. The genital area should be dried after urinating or defecating to prevent dampness.

   ☐ Strongly Agree ☐ Agree ☐ Undecided ☐ Disagree ☐ Strongly Disagree

8. The genital area should be cleaned every time a sanitary pad is changed.

   ☐ Strongly Agree ☐ Agree ☐ Undecided ☐ Disagree ☐ Strongly Disagree

9. Cleaning the genital area with clean water alone, without special soap, is sufficient.

   ☐ Strongly Agree ☐ Agree ☐ Undecided ☐ Disagree ☐ Strongly Disagree

10. Sanitary pads should be changed every 4 to 6 hours even if they are not yet full.

    ☐ Strongly Agree ☐ Agree ☐ Undecided ☐ Disagree ☐ Strongly Disagree

11. Used sanitary pads should be wrapped before being disposed of in a trash bin.

    ☐ Strongly Agree ☐ Agree ☐ Undecided ☐ Disagree ☐ Strongly Disagree

12. Used sanitary pads should not be disposed of in the toilet because they can cause blockage.

    ☐ Strongly Agree ☐ Agree ☐ Undecided ☐ Disagree ☐ Strongly Disagree

13. Sanitary pads should be changed even when at school or away from home.

    ☐ Strongly Agree ☐ Agree ☐ Undecided ☐ Disagree ☐ Strongly Disagree

14. Used sanitary pads should be wrapped in an opaque or dark-colored bag before disposal.

    ☐ Strongly Agree ☐ Agree ☐ Undecided ☐ Disagree ☐ Strongly Disagree

15. Spare sanitary pads and underwear should be carried when participating in activities outside the home.

    ☐ Strongly Agree ☐ Agree ☐ Undecided ☐ Disagree ☐ Strongly Disagree

16. A supply of sanitary pads should always be available at home.

    ☐ Strongly Agree ☐ Agree ☐ Undecided ☐ Disagree ☐ Strongly Disagree

17. Menstruation lasting more than 7 days should be discussed with a parent.
☐ Strongly Agree ☐ Agree ☐ Undecided ☐ Disagree ☐ Strongly Disagree

18. Menstruation should not interfere with learning activities at school.
☐ Strongly Agree ☐ Agree ☐ Undecided ☐ Disagree ☐ Strongly Disagree

19. If menstrual blood leaks through clothing, help should be sought immediately.
☐ Strongly Agree ☐ Agree ☐ Undecided ☐ Disagree ☐ Strongly Disagree

20. There is no need to feel embarrassed about asking questions or discussing menstrual issues.
☐ Strongly Agree ☐ Agree ☐ Undecided ☐ Disagree ☐ Strongly Disagree

MENSTRUAL HYGIENE PRACTICE QUESTIONNAIRE

Instructions:

- Read each statement carefully and answer based on what you actually do during menstruation.
- Mark (X) in the appropriate response box.

1. I bathe twice a day during menstruation.

   ☐ Yes ☐ No

2. I continue to wash my hair during menstruation.

   ☐ Yes ☐ No

3. I wash my hands with soap before and after changing a sanitary pad.

   ☐ Yes ☐ No

4. I clean the genital area every time I change a sanitary pad.

   ☐ Yes ☐ No

5. I clean the genital area from front to back.

   ☐ Yes ☐ No

6. I rinse the genital area with clean water every time I change a sanitary pad.

   ☐ Yes ☐ No

7. I dry the genital area after urinating or defecating.

   ☐ Yes ☐ No

8. I change my sanitary pad every 4 to 6 hours during menstruation.

   ☐ Yes ☐ No

9. I wrap used sanitary pads before disposing of them in a trash bin.

   ☐ Yes ☐ No

10. I do not dispose of used sanitary pads in the toilet.

    ☐ Yes ☐ No

11. I carry spare sanitary pads when I am away from home.

    ☐ Yes ☐ No

12. I carry a bag for wrapping or disposing of used sanitary pads when I am away from home.

    ☐ Yes ☐ No

13. I carry hand sanitizer or soap when traveling to maintain hand hygiene.

    ☐ Yes ☐ No

14. I continue attending school as usual during menstruation.

    ☐ Yes ☐ No

15. I continue playing with friends during menstruation.

    ☐ Yes ☐ No

16. I continue to engage in light physical activity during menstruation when my condition allows.

    ☐ Yes ☐ No
